# The extremal pentagon-chain polymers with respect to permanental sum

**DOI:** 10.1038/s41598-020-74612-w

**Published:** 2020-10-15

**Authors:** Tingzeng Wu, Hongge Wang, Shanjun Zhang, Kai Deng

**Affiliations:** 1 School of Mathematics and Statistics, Qinghai Nationalities University, Xining, 810007 People’s Republic of China; 2 The Department of Information Science, Kanagawa University, Hiratsuka city, 2591293 Japan; 3 School of Mathematics of Information Science, North Minzu University, Yinchuan, 750021 People’s Republic of China

**Keywords:** Materials chemistry, Structural materials, Applied mathematics

## Abstract

The permanental sum of a graph *G* can be defined as the sum of absolute value of coefficients of permanental polynomial of *G*. It is closely related to stability of structure of a graph, and its computing complexity is #P-complete. Pentagon-chain polymers is an important type of organic polymers. In this paper, we determine the upper and lower bounds of permanental sum of pentagon-chain polymers, and the corresponding pentagon-chain polymers are also determined.

## Introduction

The *permanent* of an $n\times n$ real matrix $M=(m_{ij})$, with $i,j \in \{1,2,\ldots ,n\}$, is defined as

$$\begin{aligned} \mathrm{per}(M)=\sum _{\sigma }\prod _{i=1}^{n}m_{i\sigma (i)}, \end{aligned}$$

where the sum is taken over all permutations $\sigma$ of $\{1,2,\ldots , n\}$.

Let *A*(*G*) be an adjacency matrix of a graph *G* of order *n* with a given vertex labeling. The *permanental polynomial* of *G* is defined as

$$\begin{aligned} \pi (G,x)=\mathrm {per}(xI-A(G))=\sum _{k=0}^{n}b_{k}(G)x^{n-k} \end{aligned}$$

with $$b_0(G)=1$$.

Earlier, Kasum et al.^1^ and Merris et al.^2^ give a graphical interpretation of the coefficients of the permanental polynomial of *G* using linear subgraphs: for $1 \le k \le n$,

$$\begin{aligned} b_{k}(G)=(-1)^{k}\sum _{H \in S_k(G)}2^{c(H)}, \end{aligned}$$

where $S_k(G)$ is the collection of all linear subgraphs *H* of order *k* in *G*, and *c*(*H*) is the number of cycles in *H*. Recall that A *linear subgraph* of a graph *G* is termed as a subgraph whose components are cycles or single edges.

The *permanental sum* of *G*, denoted by *PS*(*G*), is the sum of the absolute values of all coefficients of $\pi (G,x)$, i.e.,

$$\begin{aligned} PS(G)= \sum \limits _{k=0}^{n}|b_{k}(G)|=1+\sum \limits _{k=1}^{n}\sum _{H \in S_k(G)}2^{c(H)}. \end{aligned}$$

### Background

The study of permanental polynomial of a graph in chemical literature were started by Kasum et al.^1^. They computed respectively permanental polynomials of paths and cycles, and zeroes of these polynomials. Cash^3^ investigated permanental polynomials of some chemical graphs(including benzene, *o*-biphenylene, coronene, $C_{20}$ fullerene). And he pointed out that studying the absolute values of coefficients of permanental polynomials is of interest. However, it is difficult to compute the coefficients of permanental polynomial of a graph. Up to now, only a few about the coefficients of permanental polynomials of chemical graphs and its potential applications seems to have been published^4–14^. A natural problem is researching the sum of coefficients of permanental polynomials of a chemical graph, i.e., how characterize the permanental sum of a chemical graph. There exists a peculiar chemical phenomenon which closely relate to the permanental sum. For the theoretical study of nature, there exists 271 nonisomorphic fullerenes in $C_{50}$. Up to now, only a few fullerenes in $C_{50}$ is found. In 2004, Xie et al.^15^ captured a labile fullerene $C_{50}(D_{5h})$. Tong et al.^16^ computed the permanental sums of all 271 fullerenes in $C_{50}$. They found that the permanental sum of $C_{50}(D_{5h})$ achieves the minimum among all 271 fullerenes in $C_{50}$, and they also pointed out that the permanental sum could be closely related to the stability of molecular graphs. A bad news is the computing complexity of permanental sum is #P-complete^17^. In spite of this difficulty, the studies of permanental sums have received a lot of attention from researchers in recent years. Chou et al.^18^ studied the property of $C_{70}$. Li et al.^19^ determined the extremal hexagonal chains with respect to permanental sum. Li and Wei^20^ characterized the extremal octagonal chains with respect to permanental sum. Wu and Lai^21^ study some basic properties of the permanental sum of general graphs, in particular, they pointed out that the permanental sum is closed to the Fibonacci numbers. For the background and some known results about this problem, we refer the reader to^22–25^ and the references therein.

In addition, the permanental sum is similar to Hosoya index proposed by Haruo Hosoya. *Hosoya index* of a graph *G*, denoted by *Z*(*G*), is defined as the total number of independent edge sets of *G*^26^. The Hosoya index is closely related to the boiling points of chemical graphs. Wu and Lai^21^ shown that $PS(G)\ge Z(G)$ with the equality holds if and only if *G* is a forest. These indicate that the permanental sum is likely to explain certain characteristics of chemical molecules.

Base on arguments as above, it is interesting to study the permanental sums of chemical graphs.

### The graph model of a type of organic polymers

**Figure 1:**
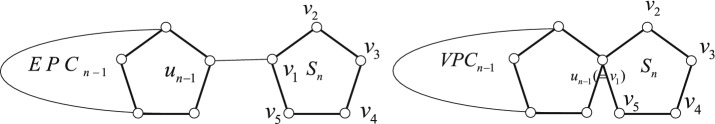
An edge-pentagon-chain $EPC_{n}$ and a vertex-pentagon-chain $VPC_{n}$.

Organic polymers are a fascinating class of chemical materials with a strikingly wide range of applications^27–32^. Many of them contain chains of five-membered rings as a building block, see Figure 1 in^33^. It is easy to see that the graph model of the organic polymer with *n* five-membered rings is an edge-pentagon-chain. An *edge-pentagon-chain* $EPC_{n}$ with *n* pentagons, which is a sub-chain of an edge-pentagon-chain, can be regarded as an edge-pentagon-chain $EPC_{n-1}$ with $n-1$ pentagons adjoining to a new terminal pentagon by a cut edge, see Fig. 1. By contracting operation of graphs, an edge-pentagon-chain $EPC_{n}$ with *n* pentagons is changed new pentagon-chain called *vertex-pentagon-chain*. That is, A *vertex-pentagon-chain*, denoted by $VPC_{n}$, is obtained by contracting every cut edge in $EPC_{n}$, see Fig. 1. Checking the structure of a vertex-pentagon-chain, it is not difficult to see that the vertex-pentagon-chain also is a graph model of a type of organic polymers^34,35^.

In this paper, we focus on properties of permanental sum of pentagon-chain polymers. We hope that results of the paper will provide theoretical support for the study of organic polymers.

### Preliminaries

Let $EPC_{n}=S_{1}S_{2}\cdots S_{n}$ be a polyomino chain with $n(\ge 2)$ pentagons, where $S_{k}$ is the *k*-th pentagon in $EPC_{n}$ attached to $S_{k-1}$ by a cut edge $u_{k-1}w_{k}$, $k=2,3,\ldots ,n$, where $w_{k}=v_{1}$ is a vertex of $S_{k}$. A vertex *v* is said to be *ortho-* and *meta-*vertex of $S_{k}$ if the distance between *v* and $w_{k}$ is 1 and 2, denoted by $o_{k}$ and $m_{k}$, respectively. Checking Fig. 1, it is easy to see that $w_{n}=v_{1}$, ortho-vertices $o_{n}=v_{2},v_{5}$, and meta-vertex $m_{n}=v_{3}, v_{4}$ in $S_{n}$.

**Figure 2:**
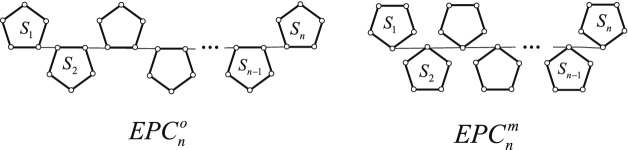
An edge-ortho-pentagon-chain $EPC^{o}_{n}$ and an edge-meta-pentagon-chain $EPC^{m}_{n}$.

**Figure 3:**
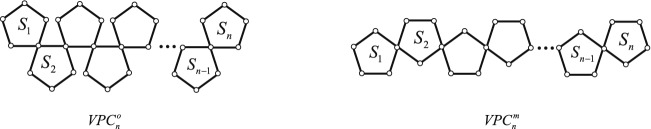
A vertex-ortho-pentagon-chain $VPC^{o}_{n}$ and a vertex-meta-pentagon-chain $VPC^{m}_{n}$.

An edge-pentagon-chain $EPC_{n}$ is an *edge-ortho-pentagon-chain* if $u_{k}=o_{k}$ for $2\le k\le n-1$, denoted by $EPC^{o}_{n}$. An edge-pentagon-chain $EPC_{n}$ is an *edge-meta-pentagon-chain* if $u_{k}=m_{k}$ for $2\le k\le n-1$, denoted by $EPC^{m}_{n}$. The resulting graphs see Fig. 2. Contracting every cut edge in $EPC^{o}_{n}$ and $EPC^{m}_{n}$, the resulting graphs are called a *vertex-ortho-pentagon-chain* $VPC^{o}_{n}$ and a *vertex-meta-pentagon-chain* $VPC^{m}_{n}$, respectively. See Fig. 3.

In^21^, some properties of permanental sum of a graph are determined.

#### **Lemma 1.1**

^21^ *Let* $P_{n}$ *be a path with n vertices. Then*

$$\begin{aligned} PS(P_{n})= {\left\{ \begin{array}{ll} 1&{} \text {if }n=0,\\ 1&{} \text {if }n=1,\\ F_{n+1}&{} \text {if }n\ge 2, \end{array}\right. } \end{aligned}$$

*where* $F(0)=0, F(1)=1$ *and* $F(n)=F(n-1)+F(n-2)$ *for* $n \ge 2$* denotes the sequence of Fibonacci numbers*.

#### **Lemma 1.2**

^21^ *The permanental sum of a graph satisfies the following identities:*

*(i ) Let G* *and H be two connected graphs. Then*

$$\begin{aligned} PS(G\cup H) = PS(G)\,PS(H). \end{aligned}$$

*(ii) Let* $e=uv$ *be an edge of a graph G and* ${{\mathcal{C}}}(e)$ *the set of cycles containing e*. *Then*

$$\begin{aligned} PS(G) = PS(G-e)+PS(G-v-u)+2\sum _{C_k \in {{\mathcal{C}}}(e)}\,PS(G-V(C_k)). \end{aligned}$$

*(iii)* *Let v* *be a vertex of a graph G and* ${{\mathcal{C}}}(v)$ *the set of cycles containing* *v*. *Then*

$$\begin{aligned} PS(G) = PS(G-v)+\sum _{u \in N_{G}(v)}PS(G-v-u)+2\sum _{C_{k} \in {{\mathcal{C}}}(v)}\,PS(G-V(C_k)). \end{aligned}$$

By Lemma 1.2, we obtain the following corollary.

#### **Corollary 1.1**

*Let G* *be a graph and v* *a vertex of G. Then* $PS(G-v )<PS(G)$.

## Results

### The bound of permanental sum of edge-pentagon-chains

**Figure 4:**
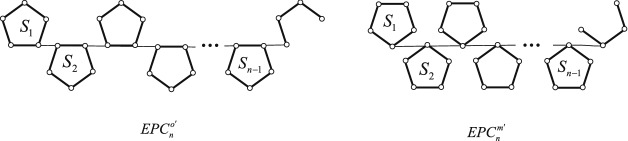
Chains $$EPC^{o'}_{n}$$ and $$EPC^{m'}_{n}$$.

In order to prove the lemma 2.1, we give two auxiliary graphs. One is denoted by $EPC^{o'}_{n}$ obtained from $EPC^{o}_{n}$ deleting a ortho-vertex in $S_{n}$. The other is denoted by $EPC^{m'}_{n}$ obtained from $EPC^{m}_{n}$ deleting meta-vertex in $S_{n}$. The resulting graphs see Fig. 4.

#### **Lemma 2.1**

*Let* $EPC^{o}_{n}$ *and* $EPC^{m}_{n}$ *be an edge-ortho-pentagon-chain and an edge-meta-pentagon-chain, respectively. Then*

$$\begin{aligned} PS(EPC^{o}_{n})& {}=  \frac{194+137\sqrt{2}}{2}\left( 8+5\sqrt{2}\right) ^{n-2}+\frac{194-137\sqrt{2}}{2}\left( 8-5\sqrt{2}\right) ^{n-2},\\ PS(EPC^{m}_{n})& {}=  \frac{640237+43067\sqrt{221}}{442}\left( \frac{15+\sqrt{221}}{2}\right) ^{n-3}+\frac{640237-43067\sqrt{221}}{442}\left( \frac{15-\sqrt{221}}{2}\right) ^{n-3}. \end{aligned}$$

#### *Proof*

By Lemma 1.2, we have

$$\begin{aligned} PS(EPC^{o}_{n})& {}=  13PS(EPC^{o}_{n-1})+5PS(EPC^{o'}_{n-1}),\\ PS(EPC^{o'}_{n})& {}=  5PS(EPC^{o}_{n-1})+3PS(EPC^{o'}_{n-1}). \end{aligned}$$

Thus,

$$\begin{aligned} \begin{pmatrix} PS(EPC^{o}_{n}) \\ PS(EPC^{o'}_{n}) \end{pmatrix} =\begin{pmatrix} 13&{} 5 \\ 5 &{} 3 \end{pmatrix} \begin{pmatrix} PS(EPC^{o}_{n-1}) \\ PS(EPC^{o'}_{n-1}) \end{pmatrix}. \end{aligned}$$

Direct computation yields $PS(EPC^{o}_{2})=194$ and $PS(EPC^{o}_{2})=80$. Now,

1 $$\begin{aligned} PS(EPC^{o}_{n})& {}=  13PS(EPC^{o}_{n-1})+5PS(EPC^{o'}_{n-1}),\nonumber \\& {} =  \begin{pmatrix} 13&5 \end{pmatrix} \begin{pmatrix} 13&{} 5 \\ 5 &{} 3 \end{pmatrix} \begin{pmatrix} PS(EPC^{o}_{n-2}) \\ PS(EPC^{o'}_{n-2}) \end{pmatrix}\nonumber \\ & {}=  \cdots \nonumber \\ & {}=  \begin{pmatrix} 13&5 \end{pmatrix} \begin{pmatrix} 13&{} 5\\ 5 &{} 3 \end{pmatrix}^{n-3} \begin{pmatrix} 194 \\ 80 \end{pmatrix}. \end{aligned}$$

Set matrix $$M= \begin{pmatrix} 13&{} 5 \\ 5 &{} 3 \end{pmatrix}.$$ Then the characteristic polynomial of *M* equals to $x^{2}-16x+14$. Solving $x^{2}-16x+14=0$, we obtain that the eigenvalues of *M* are $8+5\sqrt{2}$ and $8-5\sqrt{2}$, respectively. And the corresponding eigenvectors of these eigenvalues are $$T_{1}= \begin{pmatrix} 1\\ \sqrt{2}-1 \end{pmatrix}$$ and  $$T_{2}= \begin{pmatrix} -1\\ \sqrt{2}+1 \end{pmatrix}$$.

Let $$T= \begin{pmatrix} 1&{}-1\\ \sqrt{2}-1&{}\sqrt{2}+1 \end{pmatrix}.$$ Then the inverse matrix of *T* is $$T^{-1}=\begin{pmatrix} \frac{\sqrt{2}+2}{4}&{}\frac{\sqrt{2}}{4}\\ \frac{\sqrt{2}-2}{4}&{}\frac{\sqrt{2}}{4} \end{pmatrix}.$$ According to the property of a similarity matrix, we have

$$\begin{aligned} T^{-1}MT= \begin{pmatrix} 8+5\sqrt{2}&{}0\\ 0&{}8-5\sqrt{2} \end{pmatrix}. \end{aligned}$$

Therefore,

2 $$\begin{aligned} M=T \begin{pmatrix} 8+5\sqrt{2}&{}0\\ 0&{}8-5\sqrt{2} \end{pmatrix}T^{-1}. \end{aligned}$$

By (1) and (2), we have

$$ \begin{aligned}   PS(EPC_{n}^{o} ) &  = (\begin{array}{*{20}c}    {13} & 5  \\   \end{array} )\left( {\begin{array}{*{20}c}    1 & { - 1}  \\    {\sqrt 2  - 1} & {\sqrt 2  + 1}  \\   \end{array} } \right)\left( {\begin{array}{*{20}c}    {8 + 5\sqrt 2 } & 0  \\    0 & {8 - 5\sqrt 2 }  \\   \end{array} } \right)^{{n - 3}} \left( {\begin{array}{*{20}c}    {\frac{{\sqrt 2  + 2}}{4}} & {\frac{{\sqrt 2 }}{4}}  \\    {\frac{{\sqrt 2  - 2}}{4}} & {\frac{{\sqrt 2 }}{4}}  \\   \end{array} } \right)\left( {\begin{array}{*{20}c}    {194}  \\    {80}  \\   \end{array} } \right) \\     &  = \left( {\frac{{194 + 137\sqrt 2 }}{2}} \right)(8 + 5\sqrt 2 )^{{n - 2}}  + \left( {\frac{{194 - 137\sqrt 2 }}{2}} \right)(8 - 5\sqrt 2 )^{{n - 2}} . \\  \end{aligned} $$

Similarly, by Lemma 1.2, we obtain

$$\begin{aligned} PS(EPC^{m}_{n})&=  {} 13PS(EPC^{m}_{n-1})+5PS(EPC^{m'}_{n-1}),\\ PS(EPC^{m'}_{n})&=  {} 5PS(EPC^{m}_{n-1})+2PS(EPC^{m'}_{n-1}). \end{aligned}$$

So,

$$\begin{aligned} \begin{pmatrix} PS(EPC^{m}_{n}) \\ PS(EPC^{m'}_{n}) \end{pmatrix} =\begin{pmatrix} 13&{} 5 \\ 5 &{} 2 \end{pmatrix} \begin{pmatrix} PS(EPC^{m}_{n-1}) \\ PS(EPC^{m'}_{n-1}) \end{pmatrix}. \end{aligned}$$

Direct computation yields $PS(EPC^{m}_{2})=194$ and $PS(EPC^{m}_{2})=75$. Then,

3 $$\begin{aligned} PS(EPC^{m}_{n})& {} =  13PS(EPC^{m}_{n-1})+5PS(EPC^{m'}_{n-1})\nonumber \\ & {}=  \begin{pmatrix} 13&5 \end{pmatrix} \begin{pmatrix} 13&{} 5 \\ 5 &{} 2 \end{pmatrix} \begin{pmatrix} PS(EPC^{m}_{n-2}) \\ PS(EPC^{m'}_{n-2}) \end{pmatrix}\nonumber \\ & {} =  \cdots \nonumber \\= & {} \begin{pmatrix} 13&5 \end{pmatrix} \begin{pmatrix} 13&{} 5\\ 5 &{} 2 \end{pmatrix}^{n-3} \begin{pmatrix} 194 \\ 75 \end{pmatrix}. \end{aligned}$$

Let $$M= \begin{pmatrix} 13&{} 5 \\ 5 &{} 2 \end{pmatrix}$$ be a matrix. Then the eigenvalues of *M* are $\frac{15+\sqrt{221}}{2}$ and $\frac{15-\sqrt{221}}{2}$, respectively. And the corresponding eigenvectors of these eigenvalues are $$ T_{1}=\begin{pmatrix} 11+\sqrt{221}\\ 10 \end{pmatrix}$$ and $$ T_{2}=\begin{pmatrix} 11-\sqrt{221}\\ 10 \end{pmatrix}.$$

Let $$T=\begin{pmatrix} 11+\sqrt{221}&{}11-\sqrt{221}\\ 10&{}10 \end{pmatrix}$$. Then the inverse matrix of *T* is $$ T^{-1}=\begin{pmatrix} \frac{\sqrt{221}}{442}&{}\frac{221-11\sqrt{221}}{4420}\\ -\frac{\sqrt{221}}{442}&{}\frac{221+11\sqrt{221}}{4420} \end{pmatrix}$$. By the property of a similarity matrix, we have

$$\begin{aligned} T^{-1}MT=\begin{pmatrix} \frac{15+\sqrt{221}}{2}&{}0\\ 0&{}\frac{15-\sqrt{221}}{2} \end{pmatrix}. \end{aligned}$$

So,

4 $$\begin{aligned} M=T\begin{pmatrix} \frac{15+\sqrt{221}}{2}&{}0\\ 0&{}\frac{15+\sqrt{221}}{2} \end{pmatrix}T^{-1}. \end{aligned}$$

By (3) and (4), we have

$$\begin{aligned} PS(EPC^{m}_{n}) =\frac{640237 +43067\sqrt{221}}{442}\left( \frac{15+\sqrt{221}}{2}\right) ^{n-3} +\frac{640237 -43067\sqrt{221}}{442}\left( \frac{15-\sqrt{221}}{2}\right) ^{n-3}. \end{aligned}$$

$$\square $$

#### **Definition 2.1**

**Figure 5:**
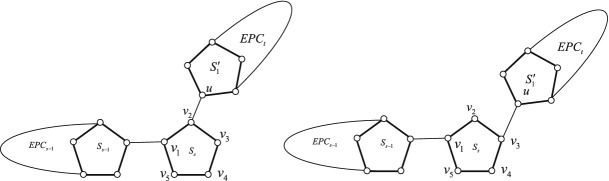
Two edge-pentagon-chains $EPC_{s} \bigotimes \limits ^{o} EPC_{t}$ and $EPC_{s} \bigotimes \limits ^{m} EPC_{t}$.

Let $EPC_{s}=S_{1}S_{2}\ldots S_{s}(s>1)$ and $EPC_{t}=S'_{1}S'_{2}\ldots S'_{t}$ be two edge-pentagon-chains. Suppose that $S_{s}=v_{1}v_{2}v_{3}v_{4}v_{5}$ in $EPC_{s}$ and *u* is a vertex of $S'_{1}$ in $EPC_{t}$. $EPC_{s} \bigotimes \limits ^{o} EPC_{t}$ is an edge-pentagon-chain obtained by attaching vertex *u* of $S'_{1}$ in $EPC_{t}$ to a ortho-vertex of $S_{s}$ in $EPC_{s}$. $EPC_{s} \bigotimes \limits ^{m} EPC_{t}$ is also an edge-pentagon-chain obtained by attaching vertex *u* of $S'_{1}$ in $EPC_{t}$ to a meta-vertex of $S_{s}$ in $EPC_{s}$. The resulting graphs see Fig. 5. We designate the transformation from $EPC_{s} \bigotimes \limits ^{m} EPC_{t}$ to $EPC_{s} \bigotimes \limits ^{o} EPC_{t}$ as type **I**.

#### **Theorem 2.1**

*Let* $EPC_{s} \bigotimes \limits ^{m} EPC_{t}$ *and* $EPC_{s} \bigotimes \limits ^{o} EPC_{t}$ *be two edge-pentagon-chains defined in Definition* 2.1. *Then*

$$\begin{aligned} PS(EPC_{s} \bigotimes \limits ^{o} EPC_{t})>PS(EPC_{s} \bigotimes \limits ^{m} EPC_{t}). \end{aligned}$$

#### *Proof*

Let $w \in V(EPC_{s-1})$ be the neighbor of $v_{1}$ in $EPC_{s}$. By Lemma 1.2, we obtain that

$$\begin{aligned}&PS(EPC_{s} \bigotimes ^{o} EPC_{t})\\&\quad =PS(EPC_{s-1})[PS(C_{5})PS(EPC_{t})+PS(P_{4})PS(EPC_{t}-u)]\\&\qquad +\,PS(EPC_{s-1}-w)[PS(P_{4})PS(EPC_{t})+PS(P_{3})PS(EPC_{t}-u)]\\&\quad =13PS(EPC_{s-1})PS(EPC_{t})+5PS(EPC_{s-1})PS(EPC_{t}-u)\\&\qquad +\,5PS(EPC_{s-1}-w)PS(EPC_{t})+3PS(EPC_{s-1}-w)PS(EPC_{t}-u) \end{aligned}$$

and

$$\begin{aligned}&PS(EPC_{s} \bigotimes ^{m} EPC_{t})\\&\quad =PS(EPC_{s-1})[PS(C_{5})PS(EPC_{t})+PS(P_{4})PS(EPC_{t}-u)]\\&\qquad +\,PS(EPC_{s-1}-w)[PS(P_{4})PS(EPC_{t})+PS(P_{1})PS(P_{2})PS(EPC_{t}-u)]\\&\quad =13PS(EPC_{s-1})PS(EPC_{t})+5PS(EPC_{s-1})PS(EPC_{t}-u)\\&\qquad +\,5PS(EPC_{s-1}-w)PS(EPC_{t})+2PS(EPC_{s-1}-w)PS(EPC_{t}-u). \end{aligned}$$

Thus $$ PS(EPC_{s} \bigotimes \limits ^{o} EPC_{t})-PS(EPC_{s} \bigotimes \limits ^{m} EPC_{t})=PS(EPC_{s-1}-w)PS(EPC_{t}-u)>0 $$. $$\square $$

Let $\mathscr {G}_{n}$ be a collection of all edge-pentagon-chains $EPC_{n}$ with *n* pentagons.

#### **Theorem 2.2**

*Let* $G\in \mathscr {G}_{n}$ *be an edge-pentagon-chain with* $n\ge 3$ *pentagons. Then*

$$\begin{aligned}&\frac{640237+43067\sqrt{221}}{442}\left( \frac{15+\sqrt{221}}{2}\right) ^{n-3}+\frac{640237-43067\sqrt{221}}{442}\left( \frac{15-\sqrt{221}}{2}\right) ^{n-3}\le PS(G)\\&\quad \le \frac{194+137\sqrt{2}}{2}\left( 8+5\sqrt{2}\right) ^{n-2}+\frac{194-137\sqrt{2}}{2}\left( 8-5\sqrt{2}\right) ^{n-2}, \end{aligned}$$

*where the first equality holds if and only if* $G\cong EPC^{m}_{n}$*, and the second equality holds if and only if* $G\cong EPC^{o}_{n}$.

#### *Proof*

Let $G=S_{1}S_{2}\ldots S_{n}\in \mathscr {G}_{n}$ be the edge-pentagon-chain with the smallest permanental sum. We show that $G= EPC^{m}_{n}$. Suppose to the contrary that $G\ne EPC^{m}_{n}$. Then there must exist $i\in (1,2,\ldots ,n)$ such that $G=EPC_{i} \bigotimes \limits ^{o} EPC_{n-i}$. By Theorem 2.1, there exists $G'=EPC_{i} \bigotimes \limits ^{m} EPC_{n-i}$ such that $PS(G')<PS(G)$, which contradicts the hypothesis *G* attains the minimum permanental sum. Thus, $G= EPC^{m}_{n}$.

Similarly, let $G=S_{1}S_{2}\ldots S_{n}\in \mathscr {G}_{n}$ be the edge-pentagon-chain with the largest permanental sum. The following we prove that $G= EPC^{o}_{n}$. Suppose to the contrary that $G\ne EPC^{o}_{n}$. Then there must exist $i\in (1,2,\ldots ,n)$ such that $G=EPC_{i} \bigotimes \limits ^{m} EPC_{n-i}$. By Theorem 2.1, there exists $G'=EPC_{i} \bigotimes \limits ^{o} EPC_{n-i}$ such that $PS(G')>PS(G)$, which contradicts the hypothesis *G* attains the maximum permanental sum. Thus, $G= EPC^{o}_{n}$.

By Lemma 2.1 and argument as above, direct yields Theorem 2.2. $\square$

### The bound of permanental sum of vertex-pentagon-chains

**Figure 6:**
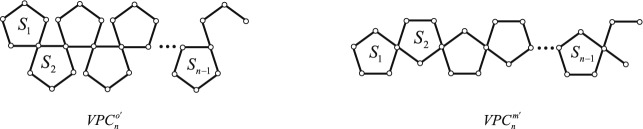
A vertex-ortho-pentagon-chain $VPC^{o'}_{n}$ and a vertex-meta-pentagon-chain $VPC^{m'}_{n}$.

We first present two auxiliary graphs. One is denoted by $VPC^{o'}_{n}$ obtained from $VPC^{o}_{n}$ deleting a ortho-vertex in $S_{n}$. The other is denoted by $VPC^{m'}_{n}$ obtained from $VPC^{m}_{n}$ deleting meta-vertex in $S_{n}$. The resulting graphs see Fig. 6.

#### **Lemma 2.2**

*Let* $VPC^{o}_{n}$ *and* $VPC^{m}_{n}$ *be a vertex-meta-pentagon-chain and a vertex-orth-pentagon-chain, respectively. Then*

$$\begin{aligned}PS(VPC^{o}_{n})=\frac{1575+157\sqrt{105}}{30}\left( \frac{7+\sqrt{105}}{2}\right) ^{n-2}+\frac{1575-157\sqrt{105}}{30} \left( \frac{7-\sqrt{105}}{2}\right) ^{n-2},\\ PS(VPC^{m}_{n})=\frac{14501+3517\sqrt{17}}{34}\left( 4+\sqrt{17}\right) ^{n-3}+\frac{145 01-3517\sqrt{17}}{34}\left( 4-\sqrt{17}\right) ^{n-3}. \end{aligned}$$

#### *Proof*

By Lemma 1.2, we have

$$\begin{aligned} PS(VPC^{o}_{n})& {} =  5PS(VPC^{o}_{n-1})+8PS(VPC^{o'}_{n-1}),\\ PS(VPC^{o'}_{n})& {}=  3PS(VPC^{o}_{n-1})+2PS(VPC^{o'}_{n-1}). \end{aligned}$$

Thus,

$$\begin{aligned} \begin{pmatrix} PS(VPC^{o}_{n}) \\ PS(VPC^{o'}_{n}) \end{pmatrix} =\begin{pmatrix} 5&{} 8 \\ 3 &{} 2 \end{pmatrix} \begin{pmatrix} PS(VPC^{o}_{n-1}) \\ PS(VPC^{o'}_{n-1}) \end{pmatrix}. \end{aligned}$$

Direct computation yields $PS(VPC^{o}_{2})=105$ and $PS(VPC^{o}_{2})=49$. Now,

5 $$\begin{aligned} PS(VPC^{o}_{n})& {} =  5PS(VPC^{o}_{n-1})+8PS(VPC^{o'}_{n-1}),\nonumber \\& {} =  \begin{pmatrix} 5&8 \end{pmatrix} \begin{pmatrix} 5&{} 8 \\ 3 &{} 2 \end{pmatrix} \begin{pmatrix} PS(VPC^{o}_{n-2}) \\ PS(VPC^{o'}_{n-2}) \end{pmatrix}\nonumber \\& {} =  \cdots \nonumber \\ & {} =  \begin{pmatrix} 5&8 \end{pmatrix} \begin{pmatrix} 5&{} 8\\ 3 &{}2 \end{pmatrix}^{n-3} \begin{pmatrix} 105 \\ 49 \end{pmatrix}. \end{aligned}$$

Set matrix $$M= \begin{pmatrix} 5&{} 8 \\ 3 &{} 2 \end{pmatrix}.$$ Then the eigenvalues of *M* are $\frac{7+\sqrt{105}}{2}$ and $\frac{7-\sqrt{105}}{2}$, respectively. And the corresponding eigenvectors of these eigenvalues are $$T_{1}= \begin{pmatrix} 16\\ \sqrt{105}-3 \end{pmatrix}$$ and  $$T_{2}= \begin{pmatrix} -16\\ \sqrt{105}+3 \end{pmatrix}$$.

Let $$T= \begin{pmatrix} 16&{}-16\\ \sqrt{105}-3&{}\sqrt{105}+3 \end{pmatrix}.$$ Then the inverse matrix of *T* is $$T^{-1}=\begin{pmatrix} \frac{\sqrt{105}+35}{1120}&{}\frac{\sqrt{105}}{210}\\ \frac{\sqrt{105}-35}{1120}&{}\frac{\sqrt{105}}{210} \end{pmatrix}.$$ According to the property of a similarity matrix, we have

$$\begin{aligned} T^{-1}MT= \begin{pmatrix} \frac{7+\sqrt{105}}{2}&{}0\\ 0&{}\frac{7-\sqrt{105}}{2} \end{pmatrix}. \end{aligned}$$

So,

6 $$\begin{aligned} M=T \begin{pmatrix} \frac{7+\sqrt{105}}{2}&{}0\\ 0&{}\frac{7-\sqrt{105}}{2} \end{pmatrix}T^{-1}. \end{aligned}$$

By (5) and (6), we have

$$\begin{aligned}&PS(VPC^{o}_{n}) =\frac{1575 +157\sqrt{105}}{30}\left( \frac{7+\sqrt{105}}{2}\right) ^{n-2}+\frac{1575 -157\sqrt{105}}{30}\left( \frac{7-\sqrt{105}}{2}\right) ^{n-2}. \end{aligned}$$

Similarly, by Lemma 1.2, we obtain

$$\begin{aligned} PS(VPC^{m}_{n})& {} =  5PS(VPC^{m}_{n-1})+8PS(VPC^{m'}_{n-1}),\\ PS(VPC^{m'}_{n})& {} =  2PS(VPC^{m}_{n-1})+3PS(VPC^{m'}_{n-1}). \end{aligned}$$

So,

$$\begin{aligned} \begin{pmatrix} PS(VPC^{m}_{n}) \\ PS(VPC^{m'}_{n}) \end{pmatrix} =\begin{pmatrix} 5&{} 8 \\ 2 &{} 3 \end{pmatrix} \begin{pmatrix} PS(VPC^{m}_{n-1}) \\ PS(VPC^{m'}_{n-1}) \end{pmatrix}. \end{aligned}$$

Direct computation yields $PS(VPC^{m}_{2})=105$ and $PS(VPC^{m}_{2})=41$. Then

7 $$\begin{aligned} PS(VPC^{m}_{n})& {} =  5PS(VPC^{m}_{n-1})+8PS(VPC^{m'}_{n-1})\nonumber \\& {} =  \begin{pmatrix} 5&8 \end{pmatrix} \begin{pmatrix} 5&{} 8 \\ 2 &{} 3 \end{pmatrix} \begin{pmatrix} PS(VPC^{m}_{n-2}) \\ PS(VPC^{m'}_{n-2}) \end{pmatrix}\nonumber \\& {} =  \cdots \nonumber \\& {} =  \begin{pmatrix} 5&8 \end{pmatrix} \begin{pmatrix} 5&{} 8\\ 2 &{} 3 \end{pmatrix}^{n-3} \begin{pmatrix} 105 \\ 41 \end{pmatrix}. \end{aligned}$$

Let $$M= \begin{pmatrix} 5&{} 8 \\ 2 &{} 3 \end{pmatrix}$$ be a matrix. Then the eigenvalues of *M* are $4+\sqrt{17}$ and $4-\sqrt{17}$, respectively. And the corresponding eigenvectors of these eigenvalues are $$ T_{1}=\begin{pmatrix} 1+\sqrt{17}\\ 2 \end{pmatrix}$$ and $$ T_{2}=\begin{pmatrix} 1-\sqrt{17}\\ 2 \end{pmatrix}.$$

Let $$T=\begin{pmatrix} 1+\sqrt{17}&{}1-\sqrt{17}\\ 2&{}2 \end{pmatrix}$$. Then the inverse matrix of *T* is $$ T^{-1}=\begin{pmatrix} \frac{\sqrt{17}}{34}&{}\frac{17-\sqrt{17}}{68}\\ -\frac{\sqrt{17}}{34}&{}\frac{17+\sqrt{17}}{68} \end{pmatrix}$$. By the property of a similarity matrix, we have

$$\begin{aligned} T^{-1}MT=\begin{pmatrix} 4+\sqrt{17}&{}0\\ 0&{}4-\sqrt{17} \end{pmatrix}. \end{aligned}$$

Therefore,

8 $$\begin{aligned} M=T\begin{pmatrix} 4+\sqrt{17}&{}0\\ 0&{}4-\sqrt{17} \end{pmatrix}T^{-1}. \end{aligned}$$

By (7) and (8), we have

$$\begin{aligned} PS(VPC^{m}_{n}) =\frac{14501 +3517\sqrt{17}}{34}\left( 4+\sqrt{17}\right) ^{n-3} +\frac{14501 -3517\sqrt{17}}{34}\left( 4-\sqrt{17}\right) ^{n-3}. \end{aligned}$$

$$\square $$

#### **Definition 2.2**

**Figure 7:**
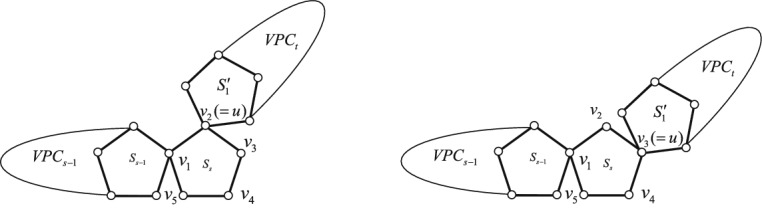
Two vertex-pentagon-chains $VPC_{s} \bigotimes \limits ^{o} VPC_{t}$ and $VPC_{s} \bigotimes \limits ^{m} VPC_{t}$.

*Let* $VPC_{s}=S_{1}S_{2}\ldots S_{s}(s>1)$ *and* $VPC_{t}=S'_{1}S'_{2}\ldots S'_{t}$ *be two vertex-pentagon-chains. Suppose that* $S_{s}=v_{1}v_{2}v_{3}v_{4}v_{5}$ *in* $VPC_{s}$ *and u is a vertex of* $S'_{1}$ *in* $VPC_{t}$. $VPC_{s} \bigotimes \limits ^{o} VPC_{t}$ *is a vertex-pentagon-chain obtained by splicing vertex u of* $S'_{1}$ *in* $VPC_{t}$ *to a ortho-vertex of* $S_{s}$ *in* $VPC_{s}$. $VPC_{s} \bigotimes \limits ^{m} VPC_{t}$ *is also a vertex-pentagon-chain obtained by splicing vertex u of* $S'_{1}$ *in* $VPC_{t}$ *to a meta-vertex of* $S_{s}$ in $VPC_{s}$.* The resulting graphs see Fig*. 7. *We designate the transformation from* $VPC_{s} \bigotimes \limits ^{m} VPC_{t}$ *to* $VPC_{s} \bigotimes \limits ^{o} VPC_{t}$ *as type* **II**.

#### **Theorem 2.3**

*Let* $VPC_{s} \bigotimes \limits ^{m} VPC_{t}$ *and* $VPC_{s} \bigotimes \limits ^{o} VPC_{t}$ *be two vertex-pentagon-chains defined in Definition* 2.2.* Then*

$$\begin{aligned} PS(VPC_{s} \bigotimes \limits ^{o} VPC_{t})>PS(VPC_{s} \bigotimes \limits ^{m} VPC_{t}). \end{aligned}$$

#### *Proof*

Let $w_{1}, w_{2} \in V(S'_{1})$ be two neighbors of *u* in $VPC_{t}$. By Lemma 1.2, we obtain that

$$\begin{aligned} PS(VPC_{s} \bigotimes ^{o} VPC_{t})& {} =  PS(VPC_{s}-v_{2})PS(VPC_{t}-u)+PS(VPC_{s}-v_{2})[PS(VPC_{t}\\&\quad -\,u-w_{1})+PS(VPC_{t}-u-w_{2})] +[PS(VPC_{s}-v_{2}-v_{1})\\&\quad +\,PS(VPC_{s}-v_{2}-v_{3})]PS(VPC_{t}-u) +2PS(VPC_{s}-v_{2})\\&\quad PS(VPC_{t}-V(S'_{1}))+2PS(VPC_{s}-V(S_{s}))PS(VPC_{t}-u)\\& {} =  [5PS(VPC_{s-1})+6PS((VPC_{s-1}-v_{1}))]PS(VPC_{t}-u)\\&\quad +\,[3PS(VPC_{s-1})+2PS((VPC_{s-1}-v_{1}))][PS(VPC_{t}-u-w_{1})\\&\quad +\,PS(VPC_{t}-u-w_{2})+2PS(VPC_{t}-V(S'_{1}))]\\& \quad +\,2PS(VPC_{s}-V(S_{s}))PS(VPC_{t}-u) \end{aligned}$$

and

$$\begin{aligned} PS(VPC_{s} \bigotimes ^{m} VPC_{t})& {} =  PS(VPC_{s}-v_{3})PS(VPC_{t}-u)+PS(VPC_{s}-v_{3})[PS(VPC_{t}\\&\quad -\,u-w_{1})+PS(VPC_{t}-u-w_{2})] +[PS(VPC_{s}-v_{3}-v_{2})\\& \quad +\,PS(VPC_{s}-v_{3}-v_{4})]PS(VPC_{t}-u) +2PS(VPC_{s}-v_{3})\\&\quad PS(VPC_{t}-V(S'_{1}))+2PS(VPC_{s}-V(S_{s}))PS(VPC_{t}-u)\\& {} =  [5PS(VPC_{s-1})+6PS((VPC_{s-1}-v_{1}))]PS(VPC_{t}-u)\\&\quad +\,[2PS(VPC_{s-1})+3PS((VPC_{s-1}-v_{1}))][PS(VPC_{t}-u-w_{1})\\&\quad +\,PS(VPC_{t}-u-w_{2})+2PS(VPC_{t}-V(S'_{1}))]\\&\quad +\,2PS(VPC_{s}-V(S_{s}))PS(VPC_{t}-u). \end{aligned}$$

By Corollary 1.1 and argument as above, we have

$$\begin{aligned}{\,}&PS(VPC_{s} \bigotimes \limits ^{o} VPC_{t})-PS(VPC_{s} \bigotimes \limits ^{m} VPC_{t})\\&\quad =[PS(VPC_{s-1})-PS((VPC_{s-1}-v_{1}))][PS(VPC_{t}-u-w_{1}) +PS(VPC_{t}-u-w_{2})\\&\qquad +\,2PS(VPC_{t}-V(S'_{1}))]>0. \end{aligned}$$

$$\square $$

Let $\mathcal{G}_{n}$ be a set of consisting all $VPC_{n}$ with *n* pentagons.

#### **Theorem 2.4**

*Let* $G\in \mathcal{G}_{n}$ *be a vertex-pentagon-chain with n pentagons. Then*

$$\begin{aligned}&\frac{14501 +3517\sqrt{17}}{34}\left( 4+\sqrt{17}\right) ^{n-3} +\frac{14501 -3517\sqrt{17}}{34}\left( 4-\sqrt{17}\right) ^{n-3}\le PS(G)\\&\quad \le \frac{1575 +157\sqrt{105}}{30}\left( \frac{\sqrt{105}+7}{2}\right) ^{n-2}+\frac{1575 -157\sqrt{105}}{30}\left( \frac{\sqrt{105}-7}{2}\right) ^{n-2}, \end{aligned}$$

*where the left equality holds if and only if* $G\cong VPC^{m}_{n}$*, and the right equality holds if and only if* $G\cong VPC^{o}_{n}$.

#### *Proof*

Let $G=S_{1}S_{2}\ldots S_{n}\in \mathcal{G}_{n}$ be the vertex-pentagon-chain with the smallest permanental sum. We show that $G= VPC^{m}_{n}$. Suppose to the contrary that $G\ne VPC^{m}_{n}$. Then there must exist $i\in (1,2,\ldots ,n)$ such that $G=VPC_{i} \bigotimes \limits ^{o} VPC_{n-i}$. By Theorem 2.3, there exists $G'=VPC_{i} \bigotimes \limits ^{m} VPC_{n-i}$ such that $PS(G')<PS(G)$, which contradicts the hypothesis *G* attains the minimum permanental sum. Thus, $G= VPC^{m}_{n}$.

Similarly, let $G=S_{1}S_{2}\ldots S_{n}\in \mathscr {G}_{n}$ be the vertex-pentagon-chain with the largest permanental sum. The following we prove that $G= VPC^{o}_{n}$. Suppose to the contrary that $G\ne VPC^{o}_{n}$. Then there must exist $i\in (1,2,\ldots ,n)$ such that $G=VPC_{i} \bigotimes \limits ^{m} VPC_{n-i}$. By Theorem 2.1, there exists $G'=VPC_{i} \bigotimes \limits ^{o} VPC_{n-i}$ such that $PS(G')>PS(G)$, which contradicts the hypothesis *G* attains the maximum permanental sum. Thus, $G= VPC^{o}_{n}$.

By Lemma 2.2 and argument as above, it is straightforward to obtain Theorem 2.4. $\square$

## Discussions

Determining extremal value is an important problem in scientific research. In this paper, we characterize the tight bound of permanental sums of all edge-pentagon-chains and vertex-pentagon-chains, respectively. And the corresponding graphs are also determined. For an edge-pentagon-chain(resp. vertex-pentagon-chain), using the computing method in Lemma 2.1(resp. Lemma 2.2) can compute the permanental sum of any edge-pentagon-chain(resp. vertex-pentagon-chain). For every organic polymers, we always find a graph model corresponding it. Thus, the permanental sum of a organic polymers can be computed by the formulas in Lemma 1.2.

$C_{50}(D_{5h})$ is captured and its permanental sum achieves the minimum among all $C_{50}$. Is the phenomenon a coincidence? Does the phenomenon exist for other chemical molecular? These are very interesting problems. However, we cannot answer them. Our motivation is to determine the extremal graphs with respect to permanental sum for some type chemical graphs in this paper. In the future, we will find the answers of the problem as above.
